# Matico

**Published:** 1850-01

**Authors:** 


*Matico*—In 1839, at a meeting of the Provincial Medical and Surgical Association of York, Dr. Jeffreys of Liverpool, introduced the Matico, as a styptic. The botanical name of the plant is Piper Angustifolium, and it is a native of Peru. Soon after Dr. Jeffreys called attention to it, the Matico, as an external application, was used with success in the Dundee Infirmary, by Dr. Munro. It exerted a happy influence over a hemorrhage from "a considerable branch of the temporal artery." Compression and cold applications failed to give any relief, but the application of the leaf, *not the powder*, arrested the hemorrhage. A hemorrhage from a wound of a branch of the palmar artery was treated in the same way with equal success.

The external use of the leaf having proved highly successful, a resort was made to its internal use. Dr. Jeffreys reports the case of a patient "who had been subject for two months to excessive discharge of pure blood and coagula from the vagina, amounting to nearly a quart in a few days, occurring every ten days or a fortnight, and followed by a serous and muco-purulent discharge." The usual treatment failed, and health was restored in a few days, by the use of a wineglass full of the infusion of Matico four times daily. In another case a hemorrhage from the bowels, an infusion of Matico, in the proportion of half an ounce to the pint, of which three tablespoonfuls were taken every four or six hours, cured the patient by the use of three doses.

In hemorrhage of the bowels, during fever, Dr. Watmough states, in the Provincial Medical Journal, that the infusion of senna leaves and the matico, two drachms of each in a pint of boiling water, and taken frequently in doses of a wineglass measure, is very successful, particularly in the melæna of typhus fever.

Dr. Horne, of London, thus speaks of Matico, as an anti-hemorrhagic medicine: "A patient suffered from an alarming epistaxis, occurring spontaneously, in October last. All remedies of the usual character failed; the case was considered beyond the reach of relief, and the brothers of the patient were sent for. One of the brothers commenced the use of the Matico, and in six hours the hemorrhage ceased, after having continued for days. There is a hemorrhagic diathesis in the family, and it has descended to the children of one of the brothers."

A relative of the family was rapidly sinking towards the grave, from disease and uterine hemorrhage, the latter of which baffled the ordinary remedies. Under the use of the Matico, the bleeding ceased, and the patient recovered.

These are the testimonials to the value of the Matico, as a styptic, which we have gleaned from the European medical journals. We are far from supposing that this Peruvian plant will displace all other means for arresting hemorrhage; the utmost that we hope for it is, that it is really useful, and may take its place confidently among the means for controlling dangerous bleeding. It is due to the time-honored standards among the anti-hemorrhagic remedies, to say they have never yet failed in our hands.—*Western Journal.—Article by one of the editors, Dr. T. S. Bell.*

